# A Lower Degree of PBMC L1 Methylation Is Associated with Excess Body Weight and Higher HOMA-IR in the Presence of Lower Concentrations of Plasma Folate

**DOI:** 10.1371/journal.pone.0054544

**Published:** 2013-01-24

**Authors:** Chandrika J. Piyathilake, Suguna Badiga, Ronald D. Alvarez, Edward E. Partridge, Gary L. Johanning

**Affiliations:** 1 The Department of Nutrition Sciences, University of Alabama at Birmingham (UAB), Birmingham, Alabama, United States of America; 2 Department of Obstetrics & Gynecology, University of Alabama at Birmingham, Birmingham, Alabama, United States of America; 3 Comprehensive Cancer Center, University of Alabama at Birmingham, Birmingham, Alabama, United States of America; 4 Department of Veterinary Sciences, The University of Texas MD Anderson Cancer Center, Smithville, Texas, United States of America; University of Bonn, Institut of Experimental Hematology and Transfusion Medicine, Germany

## Abstract

**Background:**

Identification of associations between global DNA methylation and excess body weight (EBW) and related diseases and their modifying factors are an unmet research need that may lead to decreasing DNA methylation-associated disease risks in humans. The purpose of the current study was to evaluate the following; 1) Association between the degree of peripheral blood mononuclear cell (PBMC) L1 methylation and folate, and indicators of EBW, 2) Association between the degree of PBMC L1 methylation and folate, and insulin resistance (IR) as indicated by a higher homeostasis model assessment (HOMA-IR).

**Methods:**

The study population consisted of 470 child-bearing age women diagnosed with abnormal pap. The degree of PBMC L1 methylation was assessed by pyrosequencing. Logistic regression models specified indicators of EBW (body mass index–BMI, body fat–BF and waist circumference–WC) or HOMA-IR as dependent variables and the degree of PBMC L1 methylation and circulating concentrations of folate as the independent predictor of primary interest.

**Results:**

Women with a lower degree of PBMC L1 methylation and lower plasma folate concentrations were significantly more likely to have higher BMI, % BF or WC (OR = 2.49, 95% CI:1.41–4.47, *P* = 0.002; OR = 2.49, 95% CI:1.40–4.51, *P* = 0.002 and OR = 1.98, 95%  = 1.14–3.48 *P* = 0.0145, respectively) and higher HOMA-IR (OR = 1.78, 95% CI:1.02–3.13, P = 0.041).

**Conclusion:**

Our results demonstrated that a lower degree of PBMC L1 methylation is associated with excess body weight and higher HOMA-IR, especially in the presence of lower concentrations of plasma folate.

## Introduction

A growing body of evidence indicates that global DNA hypomethylation is causally involved in the development of several human diseases. Global DNA hypomethylation is primarily due to demethylation of retrotransposons, because of their high representation in the human genome. The long interspersed nucleotide elements (L1s) are the most abundant type of retrotransposon, constituting 17–25% of the human genome [1]. These elements remain heavily methylated in normal cells. L1 repetitive element methylation levels, as measured by PCR-based methods, have been shown to correlate well with overall genomic 5-methylcytosine content [2], indicating that analysis of L1 methylation serves as a surrogate measure of global DNA methylation (DNAm) and genome-wide methylation changes in cells and tissues [3]. However, because most L1s are usually truncated at the 5′ end [4], the 5′UTR of L1s contribute to proportionately less of genomes than L1. For this reason the L1 assay will detect a relatively smaller fraction of genome-wide L1 elements and fewer CpGs of the L1 promoter, which is less supportive of methylation of the 5'UTR providing a good representation of genome methylation. To our knowledge, the correlation between L1 methylation and genome-wide methylation has not been described in peripheral blood. DNAm within the promoter region of human L1 elements plays an important role in maintaining transcriptional inactivation and inhibition of transposition [5], [6]. The expression of the L1 retrotransposon is regulated by epigenetic mechanisms, which can affect downstream gene expression genetically and epigenetically [7]. Therefore, demethylation of these elements may lead to alterations in the expression of various human genes [8], [9]. L1 retrotransposons are shown to be hypomethylated in many cancers [10], [11], [12], [13], [14] suggesting potential activation of L1 in these cancers. Based on these observations, it is biologically plausible that reactivation of L1 elements through hypomethylation could potentially induce genomic instability, thus transforming these cells into a precancerous or cancerous state. In support of this, studies have shown that L1 retrotransposition is able to induce DNA damage and apoptosis in cancer cell lines and genomic instability in human cancerous tissues [15], [16]. Even though cancer is the most commonly studied disorder in relation to L1 methylation, a reduction in L1 methylation levels in the neural tissues has been associated with the development of neural tube defects (NTDs) [17].

In addition to the genome-wide hypomethylation in target tissues, results support the notion that the degree of L1 methylation in “normal” cells such as peripheral blood mononuclear cell (PBMC) derived DNA is a potential biomarker for susceptibility for cancer [18], exposure to carcinogens [19] and obesity-related diseases, such as ischemic heart disease, stroke and cardiovascular diseases [20], [21]. A negative correlation between buffy coat L1 methylation and serum vascular cell adhesion molecule-1 (VCAM-1) among subjects free of ischemic heart disease or stroke was also recently reported, but subjects with ischemic heart disease or stroke did not show any association between serum VCAM-1 and L1 hypomethylation [22]. L1 methylation in the DNA extracted from buffy coat samples of elderly men was decreased after increased exposure to black carbon (28–60 days) and sulfate (90 days) air pollutants prior to sample collection for the methylation assay [23]. The reason for L1 hypomethylation occurrence in PBMCs in these disorders is unclear. The cumulative effects of aging or inadequate exposure to one-carbon nutrients could be envisaged to play a role in lowering L1 methylation, but it has been reported that L1 methylation is not associated with age [24], so a mechanism independent of aging is likely to be responsible. The importance of L1 methylation status during pregnancy is indicated by the observation that maternal PBMC L1 hypomethylation is associated with increased risk of congenital heart defects in children [25]. A study has also revealed a high correlation in PBMC L1 methylation between mother-daughter (*r*  =  0.48, *P*  =  0.0002), suggesting for the first time that L1 methylation could be inherited by children from their parents. This study also demonstrated a strong correlation in PBMC L1 methylation levels among only father-affected son pairs diagnosed with testicular cancer (*r*  =  0.49, *P*  =  0.03), suggesting that transgenerational inheritance of an epigenetic event could be associated with disease risk [26]. Retrotransposons are thought to be resistant to epigenetic reprogramming during embryogenesis in mice [27], and this may provide a plausible mechanism for epigenetic inheritance of L1 methylation. Excess body weight (overweight or obese) in women negatively impacts most of the above mentioned disease conditions in several ways. Obesity is a known risk factor for multiple cancers in women including endometrial cancer, cervical cancer, breast cancer, and ovarian cancer [28]. The risk for some of these cancers may be mediated via hyperinsulinemia or insulin resistance found in some obese women [21].

Identification of the association between L1 methylation and EBW, as well as diseases associated with it and their modifying factors, is an unmet research need that may lead to decreasing L1 hypomethylation-associated disease risks in humans. Obesity has increased among child-bearing age women in the US over the years, and more than 50% of child-bearing age women are either overweight or obese [29]. Because obesity is a significant health condition associated with several chronic diseases in women of child-bearing age and results in adverse pregnancy outcomes, the purpose of the current study was to evaluate the following; 1) Association between the degree of PBMC L1 methylation and folate, and indicators of EBW, 2) Association between the degree of PBMC L1 methylation and folate, and insulin resistance (IR) as indicated by a higher homeostasis model assessment (HOMA-IR).

## Methods

### Ethics Statement

The study protocol and procedures were approved by the University of Alabama at Birmingham (UAB) Institutional Review Board. All study participants gave their written informed consent.

### Study Population

The present analysis is based on 470 child-bearing age women enrolled in an ongoing prospective follow-up study funded by the NCI (R01 CA105448). The study has been described in a previous publication [30]. All women were diagnosed with abnormal cervical cells in clinics of the Health Departments in Alabama and were referred for further examination at a colposcopy clinic at the UAB. All women included in this analysis participated in an interview that assessed socio-demographic variables and lifestyle risk factors (questionnaire developed by the UAB investigators) and physical activity (CDC questionnaire). Height, weight and waist circumference (WC) measurements were obtained using standard protocols. The body mass index (BMI) was calculated using the height and weight measurements (weight kg/[height m^2^]). Percentage of body fat (BF) was measured using a TANITA-bioelectrical impedance analysis instrument (Model TBF-300A), which has been shown to provide results comparable to dual-energy X-ray absorptiometry [31]. Fasting blood samples were collected from all women.

### Laboratory Methods

The fasting blood samples were collected in EDTA containing blood collection tubes and kept on ice until they were transported to the laboratory within two hours of collection. Blood samples were processed to isolate plasma and buffy coat. All samples were immediately processed and stored at −80°C in several aliquots until the assays were completed within 2–3 months of collection. Aliquots that were never subjected to freeze-thaw cycles were used to perform all assays. DNA was extracted from the buffy coats using a standard phenol-chloroform extraction method. As described below, methylation analysis of the L1 promoter (GeneBank accession no.x58075) in PBMCs was investigated using a pyrosequencing-based method.

### Bisulfite-pyrosequencing L1 analysis

Bisulfite treatment of 1 µg of DNA extracted from buffy coat was completed using the EZ DNA methylation kit (Zymo Research, CA) and the converted DNA was eluted with 30 µl TE buffer. PCR reactions were carried out using forward (5′-TTTTTTGAGTTAGGTGTGGG-3′) and reverse-biotinylated (5′-biotin-TCTCACTAAAAAATACCAAACAA-3′) primers, as described [32]. The biotinylated PCR product, purified and made single-stranded to act as a template, was annealed to the pyrosequencing primer (5′- GGGTGGGAGTGAT-3′) (0.4 µM final concentration), and then was subjected to sequencing using an automatically generated nucleotide dispensation order for sequences to be analyzed corresponding to each reaction. The pyrograms were analyzed using allele quantification (AQ) mode to determine the proportion of C/T, and hence methylated and unmethylated cytosines at the targeted position(s). The degree of methylation was evaluated at three CpG methylation sites, a small fraction of the CpG elements in the 5′ untranslated region (5′UTR) of L1 (Figure S1). Since more than one 96-well plate was required to complete the methylation assays, we took several measures to ensure that a minimum amount of variability occurred between plates. We prepared an internal control from a one-time blood draw from a volunteer in our research team. We assayed this sample at least 30 times (entire process from bisulfite conversion through pyrosequencing) to determine the mean degree of L1 methylation. We pre-determined that if the value of this control in each plate varied by > 2% from the mean, methylation results would not be accepted for all samples on that plate, and those samples would be redone. None of the internal control values on plates varied > 2%, indicating satisfactory reproducibility of the assay between plates, allowing us to compare all samples without making adjustments. In addition, we included human methylated and non-methylated DNA standards purchased from Zymo Research in the bisulphite conversion step with each assay run. Methylation values of > % and < 5% in the methylated and non-methylated DNA standards, respectively, were considered satisfactory to accept methylation data of the study samples. In addition, non-CpG cytosine residues were used as built in controls to verify bisulphite conversion of samples. Finally, the samples were placed randomly on plates and the technician who performed the methylation assay was blinded with regard to the characteristics of the patients (BMI, folate levels, etc).

### Determination of folate, glucose and insulin

Concentrations of folate (plasma and RBC), glucose and insulin were determined using protocols previously established and validated in the laboratories of Nutrition Sciences at UAB [33]. Briefly, plasma and RBC folate were assayed by the *L. casei* microbiological assay, plasma insulin with an RIA kit from Linco Research and plasma glucose with glucose oxidase reagent on a Stanbio Sirrus analyzer. HOMA-IR was determined using the following formula: fasting glucose (mg/dl) x fasting Insulin (µU/mL)/405.

### Statistical Methods

The distribution of women by indicators of EBW were determined using standard cut points (BMI ≥25 kg/m2 and <25 kg/m2, BF ≥33% and <33% and WC ≥88 cm and <88 cm). Univariate analyses were used to compare the distribution of covariates (self–reported race, level of education, physical activity, smoking status, alcohol consumption, parity and use of oral/hormonal contraceptives) by the indicators of EBW. Pearson's chi square was used to test the differences in the categorical variables and Wilcoxon rank sum test to test differences in the median of the continuous variable. We also determined the distribution of the EBW indicators, PBMC L1 methylation, plasma folate, RBC folate and HOMA-IR using distribution plots. The normality of the distribution was tested using the Shapiro-Wilk test. The mean and the median percentage of PBMC L1 methylation by the categories of EBW and by different categories of circulating folate concentrations were determined and the difference in the medians was tested using Wilcoxon rank sum test. Unconditional logistic regression models were used to determine the association between indicators of EBW as dependent variable specified as a binary outcome, BMI (≥25 vs. <25 kg/m^2^), BF (≥33 vs. <33%) and WC (≥88 vs. <88 cm) and the degree of PBMC L1 methylation (≥median vs. <median) as the independent predictor of primary interest after adjusting for age, race, level of education, minutes of physical activity per week, smoking status, alcohol consumption, parity and use of oral/hormonal contraceptives.

As folate and PBMC L1 methylation are possible determinants of EBW, we tested the interaction between circulating concentrations of plasma folate and PBMC L1 methylation on EBW indicators regardless of the significance of the main effects. The change in deviance between the main effects and the interaction term, median plasma folate concentrations x median PBMC L1 methylation, was tested using the likelihood ratio test. The joint effects of plasma folate concentrations and PBMC L1 methylation was tested on an additive scale by categorizing the study population based on the median plasma folate and PBMC L1 methylation and using higher than median plasma folate concentration and higher than median PBMC L1 methylation as the referent category. Similarly, interaction between RBC folate and PBMC L1 methylation was tested. These analyses were adjusted for variables of age, race, level of education, minutes of physical activity per week, smoking status, alcohol consumption, parity and use of oral/hormonal contraceptives.

Since BMI is a useful predictor of insulin resistance, an indicator of metabolic disorder [34], [35], we tested if PBMC L1 methylation and circulating concentrations of folate (as categorical variable with median as the cut off) were independently associated with HOMA-IR, a measure of insulin resistance. We also tested to determine if there was an interaction between plasma folate and PBMC L1 methylation on HOMA-IR. We used the highest tertile as the cut off for the dependent variable HOMA-IR. The joint effect of plasma folate and PBMC L1 methylation was tested by using higher than median plasma folate and higher than median PBMC L1 methylation as the referent category. Similarly, interaction between RBC folate and PBMC L1 methylation on HOMA-IR was tested. All models were adjusted for variables previously mentioned. We evaluated the strength and precision of each association by estimating the odds ratio (OR), its 95% confidence interval (CI) and its statistical significance using Wald chi-square test of the null hypothesis that OR equals 1. All statistical analyses were conducted using SAS 9.1.3 version (SAS Institute, Inc, Cary NC).

## Results

### Demographic and lifestyle factors of the study population

The distribution of 470 women by the three indicators of EBW (BMI, % BF and WC) and the distribution of demographic and lifestyle variables by indicators of EBW are shown in Table 1. Approximately 60% of the women had EBW according to these indicators. The results demonstrated that Black women were significantly more likely to have BMI ≥25 kg/m^2^, % BF >33% or WC > 88 cm compared to White women (P<0.0001, P<0.001 and P = 0.0028). Smokers and users of oral/hormonal contraceptives were less likely to have BMI ≥25 kg/m^2^ (P = 0.0002 and <0.0001 respectively), % BF >33% (P<0.0001 and P = 0.0008 respectively) or WC > 88 cm (P = 0.002 and P = 0.0113). There was no statistical difference in the moderate physical activity of women by the categories of BMI and % BF. However, we observed differences of borderline significance in the moderate physical activity of women by categories of WC. Women with higher WC were more likely to be moderately active compared to women with lower WC (P = 0.0538). None of the other variables were significantly different by the categories of EBW.

**Table 1:** The demographic and life style factors of the study population based on indicators of EBW.[Table-fn nt101]

Demographic and lifestyle variables	BMI		P-value*	% BF		P-value*	WC		P-value*
	<25 kg/m^2^	≥25 kg/m^2^		<33%	≥33%		<88 cm	≥88 cm	
Number	189(59%)	277 (41%)		184 (40%)	281(60%)		196 (42%)	273 (58%)	
Median age (years)	22.8	23.7	0.1515	22.5	23.7	0.0633	22.9	23.4	0.6016
Race									
White	87 (46%)	71 (26%)	<0.0001	87 (47%)	71 (25%)	<0.0001	82 (42%)	78 (29%)	0.0028
Black	102 (54%)	206 (74%)		97 (53%)	210 (75%)		114 (58%)	195 (71%)	
Level of education									
High school education or higher	147 (78%)	213 (77%)	0.8234	144 (78%)	215 (77%)	0.6603	156 (80%)	207 (76%)	0.3360
Less than high school education	42 (22%)	64 (23%)		40 (22%)	66 (23%)		40 (20%)	66 (24%)	
Moderate activity/week!									
< 150 min	150 (80%)	225 (82%)	0.6651	145 (80%)	229 (82%)	0.6535	148 (77%)	228 (84%)	0.0538
≥ 150 min	37 (20%)	50 (18%)		36 (20%)	51 (18%)		45 (23%)	44 (16%)	
Smoking status									
Non-current	96 (51%)	188 (68%)	0.0002	90 (49%)	193 (69%)	<0.0001	103 (53%)	182 (67%)	0.0020
Current	93(49%)	89 (32%)		94 (51%)	88 (31%)		93 (47%)	91 (33%)	
Alcohol consumption									
No	57 (30%)	83 (30%)	0.9641	57 (30%)	83 (30%)	0.9344	58 (30%)	83 (30%)	0.8501
Yes	132 (70%)	194 (70%)		132 (70%)	194 (70%)		138 (70%)	190 (70%)	
Parity									
< 0 live births	61 (32%)	95 (34%)	0.6499	61 (33%)	94 (33%)	0.9465	63 (32%)	93 (34%)	0.6629
≥ 1 live births	128 (68%)	182 (66%)		123 (67%)	187 (67%)		133 (68%)	180 (66%)	
Oral/hormonal contraceptives									
Never user	15 (8%)	58 (21%)	<0.0001	16 (9%)	57 (21%)	0.0008	21 (11%)	53 (20%)	0.0113
Ever user	171 (92%)	216 (79%)		165 (91%)	221 (79%)		172 (89%)	217 (80%)	

EBW-excess body weight, BMI-body mass index , BF-body fat, WC-waist circumference

! CDC recommendation for moderate physical activity is 150 min/week

* P values for Pearson's chi-square test are shown for frequencies and median test for comparison of medians

### Distribution of EBW indicators, PBMC L1 methylation, circulating concentrations of folate and HOMA-IR

Figures S2, S3, S4, S5, S6, S7, and S8 show the distribution plots of EBW indicators, PBMC L1 methylation, circulating concentrations of folate and HOMA-IR with a normal curve superimposed on the histogram. The mean ± standard deviation (SD) and median measures for BMI were 28.6 ± 8.6 kg/m^2^ and 27.2 kg/m^2^ (Figure S2). The mean ± SD and median measures for % BF were 35.8 ± 10.8% and 36.7% (Figure S3). The mean ± SD and median measures for WC were 95.3 ± 19.1 cm and 92 cm (Figure S4). The mean ± SD and median measures for PBMC L1 methylation were 63.6 ± 7.6% and 61.7% (Figure S5). A bimodal distribution of the degree of PBMC L1 methylation was observed in our population of women and therefore we tested to determine if variables such as race, smoking status and use of oral contraceptives may explain this distribution. We observed that there was a higher percentage of blacks (66%) compared to whites (52%) in peak 1 and a higher percentage of whites (48%) compared to blacks (34%) in peak 2 (p = 0. 003). We have previously shown that black women were more likely to have lower PBMC L1 methylation compared to white women after adjusting for other likely predictors of PBMC L1 methylation [36]. Therefore, this may be one of the reasons why we have observed a higher percentage of individuals with lower methylation in peak 1 and a relatively higher percentage of individuals with higher methylation in peak 2. We did not observe significant differences in the distribution of L1 methylation by other variables such as smoking status, current drinking status and users of oral contraceptives. The mean ± SD and median measures for plasma folate were 12.2 ± 6.5 ng/mL and 11.3 ng/mL (Figure S6). The mean ± SD and median values for RBC folate were 598 ± 211 ng/mL and 566.9 ng/mL (Figure S7). The mean ± SD and median values for HOMA-IR were 4.4 ± 3.7 and 3.36 ng/mL (Figure S8). The Shapiro-Wilk test for goodness of fit had a p-value < 0.001 for all variables indicating that none of these variables are normally distributed.

### Mean ± SD and median degree of PBMC L1 methylation by EBW indicators

The mean ± SD and median degree of PBMC L1 methylation by EBW indicators is shown in Table 2. Women with higher BMI, higher % BF or higher WC had lower mean and median degree of PBMC L1 methylation compared to women with lower BMI, lower % BF or lower WC (P = 0.0011, P = 0.0008, P = 0.0156 respectively).

**Table 2:** Mean ± SD and median degree of PBMC L1 methylation by categories of EBW indicators and circulating concentrations of folate.[Table-fn nt104]

EBW indicators	PBMC L1 methylation		P-value*
	Mean ± SD	Median	
BMI < 25 kg/m^2^	65.0 ± 7.9	64.0	0.0011
BMI ≥ 25 kg/m^2^	62.6 ± 7.2	60.0	
% BF < 33	65.1 ± 8.0	63.9	0.0008
% BF ≥ 33	62.5 ± 7.0	60.2	
WC < 88 cm	64.8 ± 8.2	63.3	0.0156
WC ≥ 88 cm	62.7 ± 7.0	60.2	
Circulating concentrations of folate			
Plasma folate ≥11.29 ng/mL	64.5 ± 7.7	63.7	0.0102
Plasma folate < 11.29 ng/mL	62.7 ±7.4	60.0	
RBC folate ≥ 566.92 ng/mL	63.6 ± 7.2	62.6	0.8871
RBC folate < 566.92 ng/mL	63.5 ± 8.0	60.3	

PBMC L1-peripheral blood mononuclear cell long interspersed nucleotide element-1, EBW- excess body weight, BMI-body mass index, BF-body fat, WC-waist circumference

* Wilcoxon rank sum test

### Mean ± SD and median degree of PBMC L1 methylation by circulating concentrations of folate

The mean and median degree of PBMC L1 methylation by median plasma and RBC folate concentrations are shown in Table 2. The degree of PBMC L1 methylation was significantly lower among women with lower than median plasma folate compared to those with higher than median plasma folate (P = 0.0102). No significant differences in the degree of PBMC L1 methylation were observed by RBC folate concentrations.

### The association between the degree of PBMC L1 methylation and indicators of EBW after controlling for demographic and lifestyle factors

As shown in Table 3, in logistic regression models that tested the association between the degree of PBMC L1 methylation and indicators of EBW, we observed that women with a lower degree of PBMC L1 methylation were significantly more likely to have higher BMI, higher % BF or higher WC (P = 0.005; 0.017 and 0.008 respectively). Blacks were more likely to have higher BMI and % BF (P =  0.004 and 0.006 respectively). There was no significant association between race and WC. We also observed that current smokers were significantly less likely to have higher BMI, higher % BF or higher WC (P = 0.002 for all associations).

**Table 3:** The association between the degree of PBMC L1 methylation and indicators of EBW after controlling for demographic and lifestyle factors.[Table-fn nt106]

Demographic and lifestyle factors	Indicators of EBW		
	BMI, ≥25 vs <25 kg/m^2^	% BF, ≥33 vs <33%	WC, ≥88 vs <88 cm
	OR (95% CI)	OR (95% CI)	OR (95% CI)
Age (years)			
< 23	1.00	1.00	1.00
≥ 23	1.26 (0.77–2.05)	1.49 (0.91–2.43)	1.21 (0.76–1.94)
Race			
White	1.00	1.00	1.00
Black	2.05 (1.27–3.32)*	1.97 (1.22–3.19)*	1.27 (0.79–2.03)
Level of education			
High school education and higher	1.00	1.00	1.00
Less than high school education	1.72 (0.97–3.05)	1.76 (0.99–3.13)	1.66 (0.95–2.89)
Moderate activity/week			
< 150 min	1.00	1.00	1.00
≥ 150 min	0.90 (0.52–1.56)	0.91 (0.53–1.57)	0.61 (0.37–1.03)
Smoking status			
Non-current	1.00	1.00	1.00
Current	0.47 (0.29–0.76)*	0.46 (0.29–0.76)*	0.48 (0.29–0.77)*
Alcohol consumption			
No	1.00	1.00	1.00
Yes	1.20 (0.73–1.97)	1.03 (0.63–1.70)	1.17 (0.73–1.88)
Parity			
0 live birth	1.00	1.00	1.00
≥ 1 live birth	0.74 (0.43–1.25)	0.76 (0.45–1.29)	0.80 (0.48–1.33)
Oral/hormonal contraceptives			
Never user	1.00	1.00	1.00
Ever user	0.29 (0.15–0.59)*	0.37 (0.19–0.74)*	0.53 (0.29–0.98)*
PBMC L1 methylation**			
≥ 62%	1.00	1.00	1.00
< 62%	1.89 (1.21–2.94)*	1.71 (1.10–2.65)*	1.79 (1.17–2.72)*

PBMC L1-peripheral blood mononuclear cell long interspersed nucleotide element-1, EBW- excess body weight BMI-body mass index, BF-body fat, WC-waist circumference

* P < 0.05

We also tested the association between plasma and RBC folate concentrations and EBW indicators using the median plasma folate (11.29 ng/mL) and RBC folate (566.9 ng/mL) cut points after adjusting for demographic and lifestyle variables similar to the regression model presented in Table 3. There was no significant association between plasma folate and BMI, % BF or WC (OR = 0.82, 95% CI = 0.55–1.22, P = 0.3246; OR = 0.74, 95% CI = 0.49–1.11, P = 0.147; OR = 0.87, 95% CI = 0.58–1.29, P = 0.4838 respectively). There were no significant associations between median RBC folate and BMI or WC (OR = 1.41, 95% CI = 0.94–2.13, P = 0.096; OR = 1.30, 95% CI = 0.88–1.94, P = 0.1917, respectively). However, we observed a significant association between median RBC folate and % BF; i.e., women with RBC folate concentrations ≥ 566.9 ng/mL were more likely to have higher % BF (OR = 1.52, 95% CI = 1.00–2.30, P = 0.046) compared to women with RBC folate concentrations < 566.9 ng/mL. Even though most of the associations between folate and EBW indicators were not statistically significant, we noted that the direction of the association was negative with plasma folate and positive with RBC folate (data not shown as a Table).

### Interaction between circulating concentrations of folate and the degree of PBMC L1 methylation on EBW indicators

The distribution of the study population based on different combinations of median plasma (11.29 ng/mL) and RBC (566.9 ng/mL) folate and median degree of PBMC L1 methylation (62%) are shown in Table 4. The distribution of women in the various combinations of folate and PBMC L1 methylation groups were significantly different by the categories of EBW indicators. We observed a significant interaction between plasma folate and PBMC L1 methylation on BMI, % BF and WC (P_for interaction_ = 0.013, P_for interaction_  =  0.0041, P_for interaction_ = 0.0193, respectively). The interaction between RBC folate and the degree of PBMC L1 methylation on EBW indicators BMI and WC was not statistically significant. However, the interaction of RBC folate and the degree of PBMC L1 methylation on % BF was statistically significant (P_for interaction_ = 0.003). We further analyzed these interactions by categorizing the population based on the plasma and RBC folate and PBMC L1 methylation.

**Table 4:** The distribution of women by different combinations of plasma and RBC folate and the degree of PBMC L1 methylation by EBW indicators and HOMA-IR.[Table-fn nt108]

Combination of plasma/RBC folate and the degree of PBMC L1 methylation	N (%) of women by EBW indicator						HOMA-IR		
	BMI (kg/m^2^)		% BF		WC (cm)				
	< 25	≥ 25	< 33	≥ 33	< 88	≥ 88	< 4.53	≥ 4.53	
Plasma folate < 11.29 ng/mL & PBMC L1 methylation < 62%	33 (18%)	95 (36%)	31 (17%)	97 (36%)	37 (19%)	92 (35%)	74 (24%)	54 (39%)	
Plasma folate < 11.29 ng/mL & PBMC L1 methylation ≥ 62%	51 (27%)	45 (17%)	48 (27%)	47 (18%)	53 (28%)	43 (17%)	69 (23%)	27 (19%)	
Plasma folate ≥ 11.29 ng/mL & PBMC L1 methylation < 62%	41 (22%)	54 (21%)	42 (23%)	53 (20%)	41 (22%)	54 (21%)	70 (23%)	23 (17%)	
Plasma folate ≥ 11.29 ng/mL & PBMC L1 methylation ≥ 62%	61 (33%)	69 (26%)	59 (33%)	71 (26%)	60 (31%)	71 (27%)	93 (30%)	35 (25%)	
*P-value* *	0.0002		0.0002		0.0007		0.0180		
RBC folate < 566.9 ng/mL & PBMC L1 methylation < 62%	37 (19%)	81 (30%)	35 (19%)	83 (31%)	41 (22%)	79 (30%)	77 (25%)		42 (30%)
RBC folate < 566.9 ng/mL & PBMC L1 methylation ≥ 62%	62 (33%)	44 (17%)	63 (35%)	43 (16%)	60 (31%)	46 (18%)	81 (27%)		25 (18%)
RBC folate ≥ 566.9 ng/mL & PBMC L1 methylation < 62%	37 (19%)	68 (26%)	38 (21%)	67 (25%)	37 (19%)	67 (26%)	67 (22%)		35 (25%)
RBC folate ≥ 566.9 ng/mL & PBMC L1 methylation ≥ 62%	50 (27%)	70 (27%)	44 (24%)	75 (28%)	53 (28%)	68 (26%)	81 (26%)		37 (27%)
*P-value* *	0.0002		0.0001		0.0027		0.2234		

PBMC L1-peripheral blood mononuclear cell long interspersed nucleotide element-1, EBW- excess body weight BMI-body mass index, BF-body fat, WC-waist circumference

* Pearson's chi-square test

As shown in Table 5, women with plasma folate <11.29 ng/mL and PBMC L1 methylation <62% were significantly more likely to have ≥25 kg/m^2^ BMI, ≥33% BF or ≥88 cm WC compared to women with plasma folate ≥ 11.29 ng/mL and a degree of PBMC L1 methylation ≥62% (OR = 2.49, 95% CI = 1.41–4.47, P = 0.0016; OR = 2.49, 95% CI = 1.40–4.51, P = 0.0019; OR = 1.98 95% CI = 1.14–3.48, P = 0.0145, respectively). As shown in the table, p values for the interaction term were statistically significant for all three associations. In these models, Blacks were more likely to have higher BMI and % BF (OR = 2.12, 95% CI = 1.33–3.39, P = 0.002; OR = 2.07, 95% CI = 1.30–3.30, P = 0.002 respectively). There was no significant association between race and WC. Women with less than high school education were significantly more likely to have higher BMI, % BF and WC (OR =  2.19, 95% CI = 1.27–3.86, P = 0.001; OR = 2.14, 95% CI = 1.24–3.80, P = 0.006; OR = 2.11, 95% CI = 1.23–3.68, P = 0.006, respectively). We also observed that current smokers were less likely to have higher BMI, higher % BF or higher WC (OR = 0.55, 95% CI = 0.34–0.88, P = 0.0124; OR = 0.49, 95% = 0.31–0.79, P = 0.003; OR = 0.56, 95% CI = 0.35–0.88, P = 0.0127, respectively). Users of oral contraceptives were less likely to have higher BMI, higher % BF or higher WC (OR = 0.33, 95% CI = 0.17–0.62, P = 0.0004; OR = 0.41, 95% CI = 0.21–0.76, P = 0.004; OR = 0.51, 95% CI = 0.28–0.90, P = 0.02, respectively).

**Table 5:** Interaction of plasma folate and the degree of PBMC L1 methylation on indicators of EBW after adjusting for demographic and lifestyle factors.[Table-fn nt110]

Demographic and lifestyle factors	Indicators of EBW		
	BMI, ≥25 vs <25 kg/m^2^	% BF, ≥33 vs <33%	WC, ≥88 vs <88 cm
	OR (95% CI)	OR (95% CI)	OR (95% CI)
Age (years)			
< 23	1.00	1.00	1.00
≥ 23	1.45 (0.92–2.30)	1.57 (0.99–2.50)	1.32 (0.85–2.07)
Race			
White	1.00	1.00	1.00
Black	2.12 (1.33–3.39)*	2.07 (1.30–3.30)*	1.45 (0.92–2.30)
Level of education			
High school education and higher	1.00	1.00	1.00
Less than high school education	2.19 (1.27–3.86)*	2.14 (1.24–3.80)*	2.11 (1.23–3.68)*
Moderate activity/week			
< 150 min	1.00	1.00	1.00
≥ 150 min	0.98 (0.57–1.69)	0.97 (0.56–1.67)	0.60 (0.36–1.00)
Smoking status			
Non-current	1.00	1.00	1.00
Current	0.55 (0.34–0.88)*	0.49 (0.31–0.79)*	0.56 (0.35–0.88)*
Alcohol consumption			
No	1.00	1.00	1.00
Yes	1.08 (0.68–1.74)	1.02 (0.63–1.64)	1.13 (0.72–1.79)
Parity			
0 live birth	1.00	1.00	1.00
≥ 1 live birth	0.70 (0.43–1.14)	0.74 (0.45–1.22)	0.86 (0.53–1.39)
Oral/hormonal contraceptives			
Never user	1.00	1.00	1.00
Ever user	0.33 (0.17–0.62)*	0.41 (0.21–0.76)*	0.51 (0.28–0.90)*
Plasma folate and PBMC L1 methylation categories			
Plasma folate >11.29 ng/mL PBMC L1 methylation ≥ 62%	1.00	1.00	1.00
Plasma folate > 11.29 ng/mL PBMC L1 methylation < 62%	1.24 (0.70–2.20)	1.04 (0.59–1.85)	1.11 (0.63–1.95)
Plasma folate < 11.29 ng/mL PBMC L1 methylation ≥ 62%	0.70 (0.39–1.23)	0.69 (0.39–1.22)	0.68 (0.39–1.19)
Plasma folate < 11.29 ng/mL PBMC L1 methylation < 62%	2.49 (1.41–4.47)*	2.49 (1.40–4.51)*	1.98 (1.14–3.48) *
*P value for Interaction*	0.0130	0.0041	0.0193

PBMC L1-peripheral blood mononuclear cell long interspersed nucleotide element-1, EBW- excess body weight BMI-body mass index, BF-body fat, WC-waist circumference

* P < 0.05

As shown in Table 6, we observed that women with RBC folate < 566.9 ng/mL and PBMC L1 methylation ≥ 62% were less likely to have higher BMI and % BF compared to women with RBC folate ≥566.9 ng/mL and PBMC L1 methylation ≥62%; the p value for the interaction term was statistically significant for % BF (OR = 0.35, 95% CI = 0.19–0.62, P = 0.0033) and only approached statistical significance for BMI (OR = 0.49, 95% CI = 0.28–0.87, P = 0.0735).

**Table 6:** Interaction of RBC folate and the degree of PBMC L1 methylation on indicators of EBW after adjusting for demographic and lifestyle factors.[Table-fn nt112]

Demographic and lifestyle factors	Indicators of EBW		
	BMI, ≥25 vs <25 kg/m^2^	% BF, ≥33 vs <33%	WC, ≥88 vs <88 cm
	OR (95% CI)	OR (95% CI)	OR (95% CI)
Age (years)			
< 23	1.00	1.00	1.00
≥ 23	1.38 (0.87–2.20)	1.50 (0.94–2.40)	1.27 (0.81–1.98)
Race			
White	1.00	1.00	1.00
Black	2.21 (1.38–3.54)*	2.19 (1.36–3.52)*	1.49 (0.94–2.36)
Level of education			
High school education and higher	1.00	1.00	1.00
Less than high school education	2.25 (1.30–3.98)	2.25 (1.29–4.02)	2.15 (1.26–3.75)
Moderate activity/week			
< 150 min	1.00	1.00	1.00
≥ 150 min	0.98 (0.57–1.68)	0.97 (0.57–1.67)	0.60 (0.36–1.00)
Smoking status			
Non-current	1.00	1.00	1.00
Current	0.54 (0.34–0.87)*	0.48 (0.30–0.77)*	0.56 (0.35–0.88)*
Alcohol consumption			
No	1.00	1.00	1.00
Yes	1.10 (0.69–1.76)	1.02 (0.63–1.64)	1.16 (0.74–1.83)
Parity			
0 live birth	1.00	1.00	1.00
≥ 1 live birth	0.72 (0.44–1.18)	0.77 (0.46–1.26)	0.88 (0.55–1.43)
Oral/hormonal contraceptives			
Never user	1.00	1.00	1.00
Ever user	0.35 (0.18–0.65)*	0.44 (0.23–0.81)*	0.52 (0.28–0.91)*
Folate and PBMC L1 methylation categories			
RBC folate ≥ 566.9 ng/mL PBMC L1 methylation > 62%	1.00	1.00	1.00
RBC folate ≥ 566.9 ng/mL PBMC L1 methylation < 62%	1.46 (0.82–2.61)	1.04 (0.58–1.88)	1.40 (0.80–2.47)
RBC folate < 566.9 ng/mL PBMC L1 methylation > 62%	0.49 (0.28–0.87)*	0.35 (0.19–0.62)*	0.61 (0.35–1.05)
RBC Folate < 566.9 ng/mL PBMC L1 methylation < 62%	1.54 (0.86–2.78)	1.29 (0.71–2.36)	1.40 (0.81–2.54)
*P value for Interaction*	0.0735	0.0033	0.2448

PBMC L1-peripheral blood mononuclear cell long interspersed nucleotide element-1, EBW- excess body weight BMI-body mass index, BF-body fat, WC-waist circumference, RBC- red blood cell

* P < 0.05

### Association between circulating concentrations of folate, PBMC L1 methylation and HOMA-IR

Although women with a degree of PBMC L1 methylation ≥ 62% were ∼30% less likely to have HOMA-IR ≥ 4.53, the association was not statistically significant. We also observed that women with plasma folate ≥ 11.29 were ∼30% less likely to have HOMA-IR ≥ 4.53 but the association was not statistically significant. We also observed that women with RBC folate ≥ 566.9 had a tendency to have higher HOMA-IR but it was not statistically significant.

### Interaction between circulating concentrations of folate and PBMC L1 methylation on HOMA-IR

The distribution of women in different combinations of folate and the degree of PBMC L1 methylation was statistically different by HOMA-IR categories with plasma folate (P = 0.0180), but not with RBC folate (Table 4). We tested the interaction between median circulating concentration of folate and the degree of PBMC L1 methylation on HOMA-IR. As shown in Table 7, we observed a significant interaction between median plasma folate and the median degree of PBMC L1 methylation; i.e., women with plasma folate < 11.29 ng/mL and PBMC L1 methylation < 62% were more likely to have higher HOMA-IR compared to women with plasma folate ≥ 11.29 ng/mL and PBMC L1 methylation ≥ 62% (OR = 1.78, 95% CI = 1.02–3.13, P = 0.0411, P_for interaction_ = 0.0452). There was no association between different combinations of RBC folate and the degree of PBMC L1 methylation on HOMA-IR (P_for interaction_ = 0.1852) (Table 7). In these models we observed that smokers were less likely to have higher HOMA-IR and users of oral/hormonal contraceptives were more likely to have higher HOMA-IR.

**Table 7:** Effect of the interaction between plasma folate/RBC folate and the degree of PBMC L1 methylation on HOMA-IR.[Table-fn nt114]

Risk Factors	HOMA-IR (≥4.53 vs <4.53)	
	OR (95% CI)	OR (95% CI)
Age (years)		
< 23	1.00	1.00
≥ 23	1.02 (0.64–1.62)	0.97 (0.61–1.54)
Race		
White	1.00	1.00
Black	1.44 (0.87–2.40)	1.56 (0.95–2.60)
Level of education		
High school education and higher	1.00	1.00
Less than high school education	0.93 (0.52–1.63)	0.99 (0.55–1.74)
Moderate activity/week		
< 150 min	1.00	1.00
≥ 150 min	1.22 (0.71–2.07)	1.25 (0.73–2.11)
Smoking status		
Non-current	1.00	1.00
Current	0.55 (0.33–0.90)*	0.55 (0.33–0.90)*
Alcohol consumption		
No	1.00	1.00
Yes	0.72 (0.45–1.15)	0.73 (0.46–1.17)
Parity		
0 live birth	1.00	1.00
≥ 1 live birth	0.95 (0.57–1.57)	0.99 (0.60–1.64)
Oral/hormonal contraceptives		
Never user	1.00	1.00
Ever user	1.97 (1.08–3.75)*	1.94 (1.07–3.66)*
Plasma folate and PBMC L1 methylation		
Plasma Folate ≥ 11.29 ng/mL PBMC L1 methylation ≥ 62% (n = 130)	1.00	
Plasma folate ≥ 11.29 ng/mL PBMC L1 methylation < 62% (n = 95)	0.81 (0.43–1.54)	
Plasma folate < 11.29 ng/mL PBMC L1 methylation ≥ 62% (n = 96)	0.89 (0.48–1.66)	
Plasma folate < 11.29 ng/mL PBMC L1 methylation < 62% (n = 131)	1.78 (1.02–3.13)*	
*P value for Interaction*	0.0452	
RBC folate and PBMC L1 methylation		
RBC Folate ≥ 566.9 ng/mL PBMC L1 methylation ≥ 62%		1.00
RBC folate ≥ 566.9 ng/mL PBMC L1 methylation < 62%		1.05 (0.58–1.90)
RBC folate < 566.9 ng/mL PBMC L1 methylation ≥ 62%		0.55 (0.29–1.01)
RBC folate < 566.9 ng/mL PBMC L1 methylation < 62%		1.03 (0.58–1.82)
*P value for Interaction*		0.1852

PBMC L1-peripheral blood mononuclear cell long interspersed nucleotide element-1, HOMA-IR-homeostasis model assessment- insulin resistance, RBC- red blood cell

* P < 0.05

## Discussion

An improved understanding of environmental influences on epigenetic processes in humans will promote our ability to provide recommendations based on population-wide exposures, which may in turn have a significant influence on the obesity epidemic. Fortification of grain products with folic acid (FA), which was mandated by the FDA in 1998 to reduce the risk of NTDs in the US, has induced a population-wide increase in folate intake. However, our results indicated that women with plasma folate lower than 11.29 ng/ml and with lower PBMC L1 methylation are still likely to be at higher risk for EBW and insulin resistance. Even though folate plays a major role in generating methyl groups for methylation reactions, and aberrant DNAm plays a significant role in modifying several obesity associated diseases, the influence of this population-wide exposure on the methylation machinery in relation to obesity related outcomes is virtually unknown. A previous study conducted in the US post FA fortification era among an older (45–75 years) population of men and women largely with EBW demonstrated that several indicators of EBW were significant predictors for PBMC L1 methylation, but these associations did not remain significant after adjusting for age, gender and race [37].

The ideal target tissue to identify DNAm aberrations responsible for EBW could be the hypothalamus of the brain rather than PBMCs. However, there is increasing evidence to support the idea that PBMC DNA is useful to evaluate epigenetic changes, as epimutations are not limited to target tissues but can also be detected in peripheral blood cells [38], [39]. Our study conducted among a younger population (women of child-bearing age) demonstrated that a lower degree of PBMC L1 methylation was significantly associated with higher EBW after adjusting for age, race and several other confounding variables, and the results were consistent with all three indicators of EBW. To our knowledge, this is the first study to report an independent association between the degree of PBMC L1 methylation and several indicators of EBW. We noted that the observed differences in the degree of PBMC L1 methylation between women with EBW and with no EBW were relatively small (3–4%). However, significant independent associations between PBMC L1 methylation and indicators of EBW suggest that even smaller improvements in methylation are likely to have biologically meaningful effects on EBW. Our results also demonstrated that a lower degree of L1 methylation is associated with a greater risk for EBW, especially in the presence of lower folate. Since folate is one of the most important methyl donor nutrients, our observations suggest that women with lower folate status are likely to maintain L1 methylation at a lower level resulting in hypomethylation-mediated reactivation of L1s. Activated L1 have been reported to be present in the regulatory regions of many genes involved in food intake, obesity, and metabolic syndrome [40], [41], [42]. These observations support our findings of a significant influence of L1 methylation and folate on EBW and insulin resistance.

A previous study reported that a higher degree of methylation in Alu repeats in peripheral blood leukocytes was associated with higher HOMA among monozygotic twins [43]. In our study, the degree of PBMC L1 methylation was not independently associated with HOMA, but the direction of the association was opposite to that of the previous study. Even though both the degree of methylation in both Alu and LINE-1 repeats serves as a surrogate marker for estimating global DNAm levels, their differential effects on the stability of DNA or expression of specific genes, via polymorphic insertions in the human genome [44] may explain the disagreement between the previous study and ours. Our observation that women with a lower degree of PBMC L1 methylation and lower plasma folate levels were significantly more likely to have higher HOMA provide a possible link between folate, L1 methylation and risk of metabolic syndrome associated diseases. A previous study has shown that the degree of blood derived L1 methylation is positively associated with metabolic syndrome phenotypes, such as fasting glucose and plasma lipid levels [45]. To our knowledge, this is the first study to report that having a lower degree of L1 methylation along with lower plasma folate status is associated with higher HOMA.

Since both lower folate status [46] and EBW [47] appear to increase the risk for neural tube defects (NTDs), several studies including the US National Health and Nutrition Examination Surveys (NHANES) attempted to address whether there is a relationship between obesity and circulating concentrations of folate, an important question considering that > 50% of women of child-bearing age have EBW, but the results reported have been largely inconclusive. NHANES III in the pre-fortification era (1994–1998) and early post-fortification era (1999–2000) reported that obese adults compared to normal weight adults had greater odds of having low concentrations of serum folate [48], [49]. A post-fortification NHANES, which analyzed serum folate (2003–2008) and RBC folate (2007–2008), reported that BMI was inversely associated with serum folate among those who did not use supplements containing FA, but not among supplement users. This report also documented that regardless of supplement use, obese women had the highest RBC concentrations [50]. In our study, we observed that women with higher RBC folate concentrations were more likely to have EBW, but these associations were of only borderline significance. The interaction between RBC folate and L1 methylation on both EBW and HOMA differed from the interaction between plasma folate and L1 on the same variables, suggesting that folate stored in RBCs of women with EBW may not be readily available for biological functions such as L1 methylation.

In this study, we also observed an inverse association between the use of oral/hormonal contraceptives and EBW indicators. Even though weight gain is often attributed as a side effect of contraceptive use [51], there is no scientific evidence to indicate that oral contraceptives induce weight gain [52]. Our observation may be due to contraceptive users adopting “obesity-preventive” lifestyle/dietary habits because of the belief that contraceptive use may result in EBW. The inverse association between HOMA-IR and oral/hormonal contraceptive may be due to the fact that women with EBW were less likely to use oral/hormonal contraceptives. The positive association observed between alcohol consumption and HOMA-IR agrees with previous studies which reported that alcohol is considered to be a potential risk factor for the incidence of type 2 diabetes mellitus, which causes insulin resistance and pancreatic β-cell dysfunction [53]. Further investigations are required to understand the mechanisms underlying these observed associations.

A national survey in France documented that obese women were less likely to use contraceptives and had more unplanned pregnancies [54]. Further, some studies have shown that obesity negatively affects contraception [55], [56], suggesting that unplanned pregnancies are likely to be more prevalent among obese women. Because the obesity-related disease risk profiles observed in reproductive age women may reflect stable adult profiles that may present at the time of conception, concerns about their transgenerational effects have gained increased attention in recent years. These observations collectively suggest that women with EBW who plan a pregnancy may need to be counseled to participate in nutrition programs aimed to achieve optimum methylation and folate status prior to conception. Animal models have demonstrated that supplementation with methyl donors (folic acid, vitamin B12, betaine and choline) prevents transgenerational amplification of obesity, suggesting that DNAm mechanisms are involved in this process [57]. It is unclear whether similar transgenerational effects occur in humans exposed to higher levels of methyl donors.

Another interesting observation in our study is the bimodal distribution of the degree of PBMC L1 methylation. Even though a bimodal distribution has not been reported for L1 methylation, a distribution of this nature has been observed in CpG methylation detected by other techniques [58]. A bimodal distribution may not be observed in a healthy homogenous population. However, a distribution of this nature is somewhat expected in a population like ours at a risk for cervical cancer which also includes individuals with different racial groups and varying lifestyle factors such as smoking, oral contraceptives etc. In this study the bimodal distribution is most likely due to heterogeneity of the population with regard to race of the study participants. Since we have adjusted for race and have used the median cut point for L1 methylation in our models, we believe that our results are not confounded by the bimodal distribution of PBMC L1 methylation.

In conclusion, our results demonstrated that a lower degree of L1 methylation is associated with a greater risk for EBW and insulin resistance, especially in the presence of lower folate. Since folate is one of the most important methyl donor nutrients, one plausible hypothesis is that women with lower folate status are likely to maintain L1 methylation at a lower level, resulting in hypomethylation-mediated reactivation of L1s. Future studies are needed to document transgenerational effects of lower L1 methylation in relation to exposure to methyl donors. These studies are important even in the post FA fortification era since a fraction of women are still unlikely to have sufficient folate concentrations required for optimum PBMC L1 methylation. Future studies with larger sample size and longitudinal design are needed for examining a causal link between exposure to one-carbon micronutrients, L1 methylation and EBW related health outcomes.

Interpretation of our results is limited by several constraints. We make the assumption that EBW may have resulted in changes in the degree of PBMC L1 methylation, which further leads to EBW-related co-morbidities. However, because of the cross sectional nature of the study design, we are unable to determine whether the observed methylation or folate changes are the cause or the consequence of EBW. Future studies with individuals who changed their EBW status over time will be required to clarify the causal directions. Confirmation of causality will provide new insight into folate-based interventions for prevention of EBW and related diseases in the future. Finally, our results will apply only to women diagnosed with abnormal pap which consists of ∼13% of US women, indicating the need for replication of the observed results in other populations.

## Supporting Information

Figure S1 **Location of L1 pyrosequencing sites 1-3. CpG island region of the human** ***L1 transposon*** **(GenBank accession no. X58075, nucleotide position 1 to 1147).** Yellow highlights represent single CpG sites, and red highlights (Site1, Site 2 and Site 3) represent the CpG sites analyzed by pyrosequencing. Horizontal arrows indicate the location of primers (F, forward primer; R, reverse primer; -bio, biotinylated primer). The sequencing primer is underlined. The complementary strand was analyzed.
(TIFF)

Figure S2 **Distribution of BMI in the study population.**
(TIF)

Figure S3 **Distribution of % BF in the study population.**
(TIF)

Figure S4 **Distribution of waist circumference in the study population.**
(TIF)

Figure S5 **Distribution of PBMC L1 methylation in the study population.**
(TIF)

Figure S6 **Distribution of plasma folate in the study population.**
(TIF)

Figure S7 **Distribution of RBC folate in the study population.**
(TIF)

Figure S8 **Distribution of HOMA-IR in the study population.**
(TIF)
