# Supplementary figures and images for: A Lower Degree of PBMC L1 Methylation Is Associated with Excess Body Weight and Higher HOMA-IR in the Presence of Lower Concentrations of Plasma Folate
Source: PLoS One. 2013 Jan 24;8(1):e54544. doi: 10.1371/journal.pone.0054544 (PMC3554730; doi:10.1371/journal.pone.0054544)

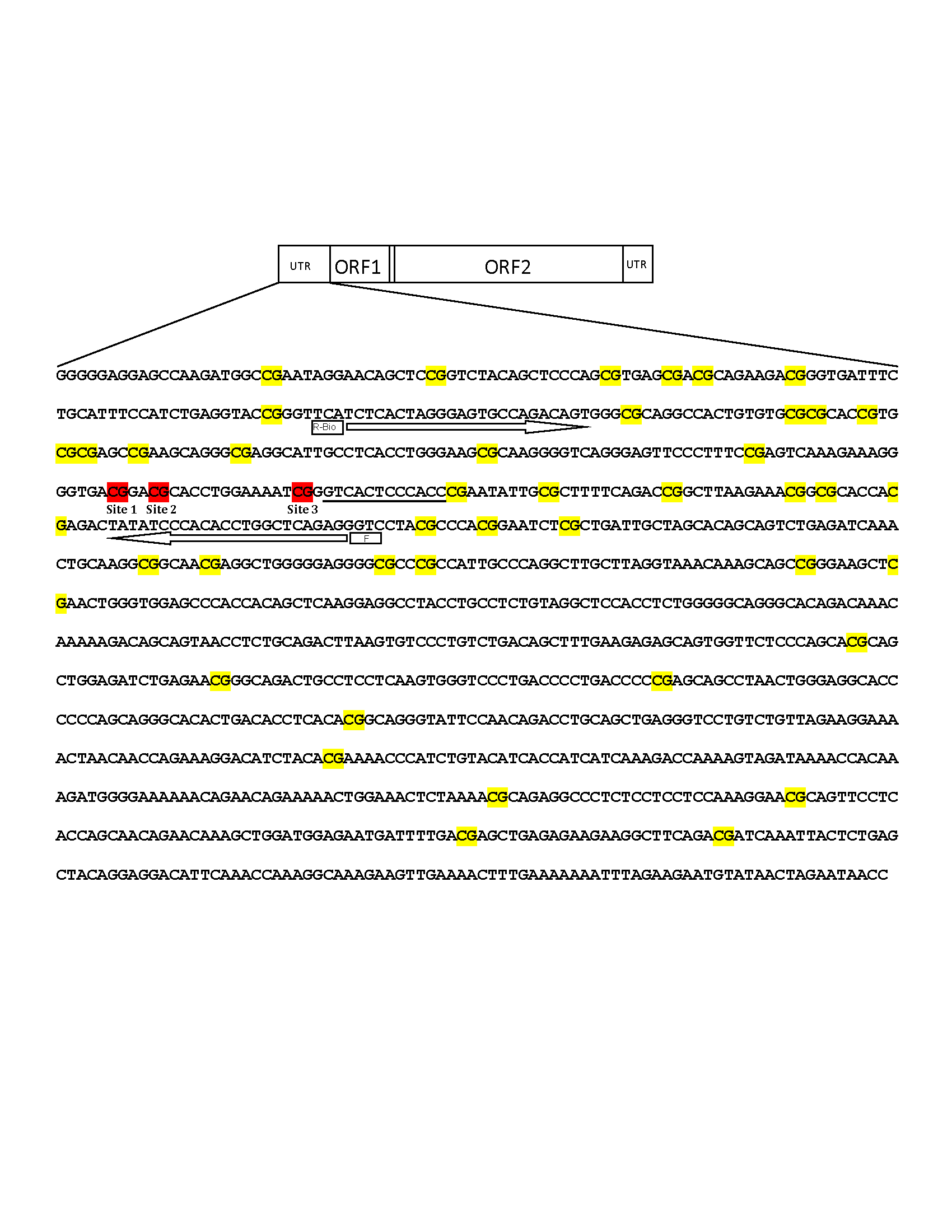

Supplement: Figure S1 — Location of L1 pyrosequencing sites 1-3. CpG island region of the human L1 transposon (GenBank accession no. X58075, nucleotide position 1 to 1147). Yellow highlights represent single CpG sites, and red highlights (Site1, Site 2 and Site 3) represent the CpG sites analyzed by pyrosequencing. Horizontal arrows indicate the location of primers (F, forward primer; R, reverse primer; -bio, biotinylated primer). The sequencing primer is underlined. The complementary strand was analyzed. (TIFF) [file pone.0054544.s001.tiff]

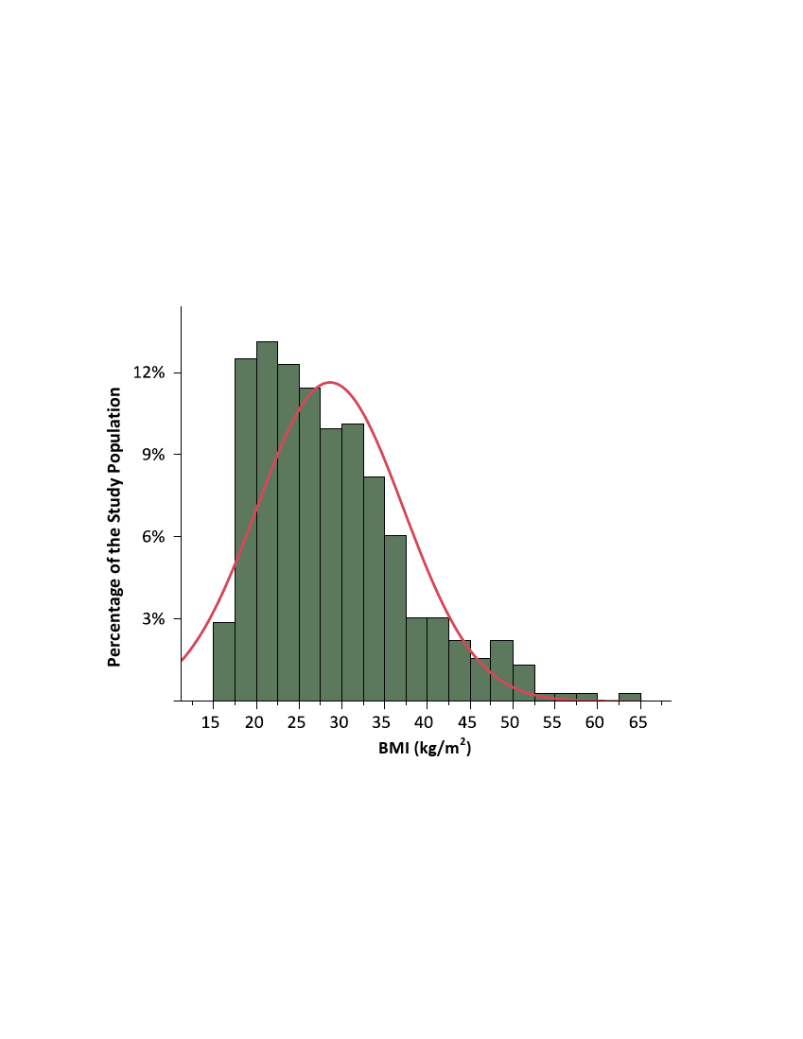

Supplement: Figure S2 — Distribution of BMI in the study population. (TIF) [file pone.0054544.s002.tif]

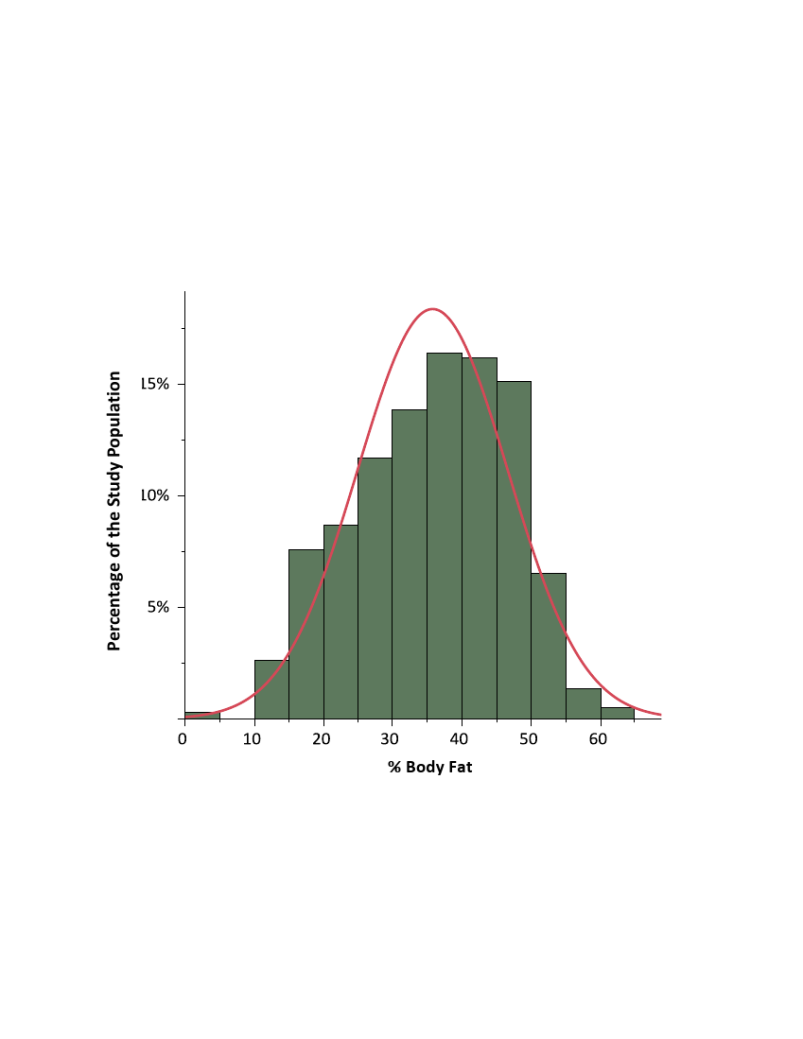

Supplement: Figure S3 — Distribution of % BF in the study population. (TIF) [file pone.0054544.s003.tif]

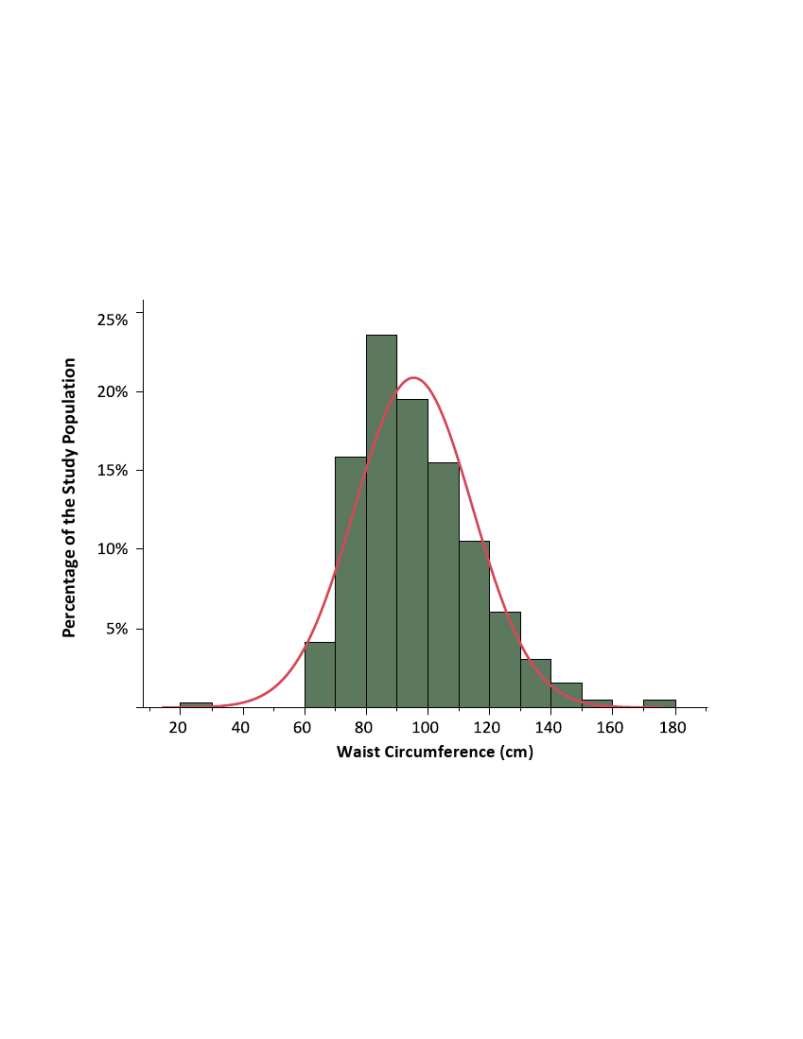

Supplement: Figure S4 — Distribution of waist circumference in the study population. (TIF) [file pone.0054544.s004.tif]

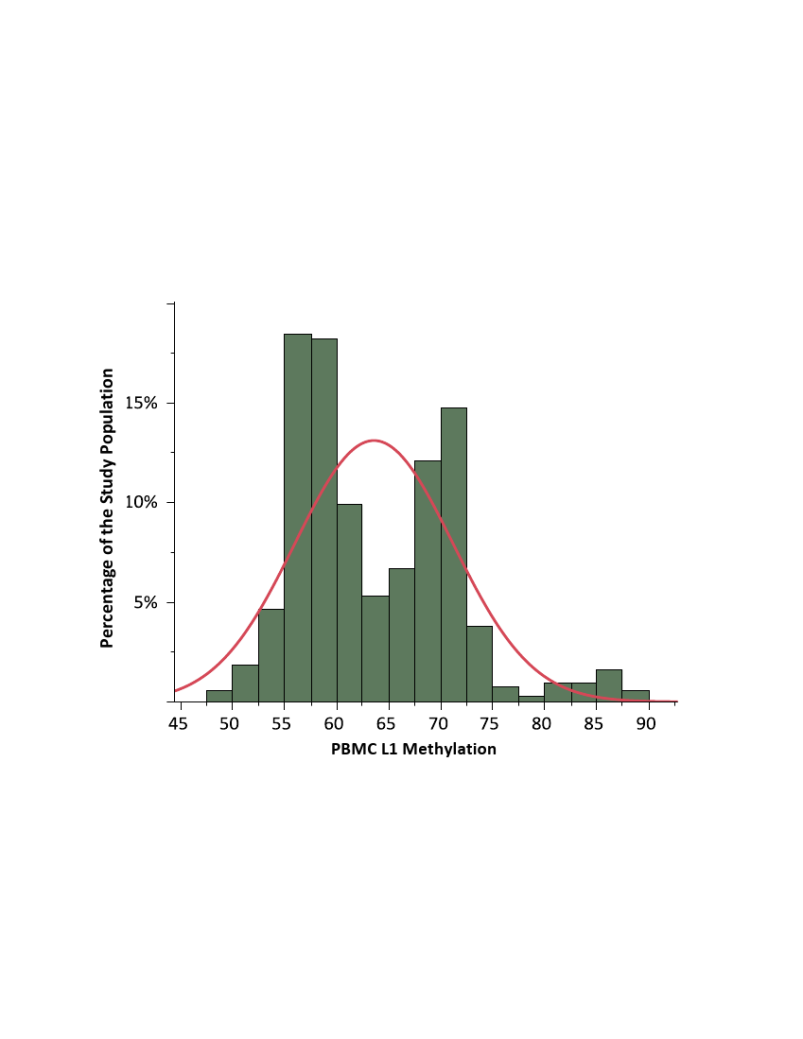

Supplement: Figure S5 — Distribution of PBMC L1 methylation in the study population. (TIF) [file pone.0054544.s005.tif]

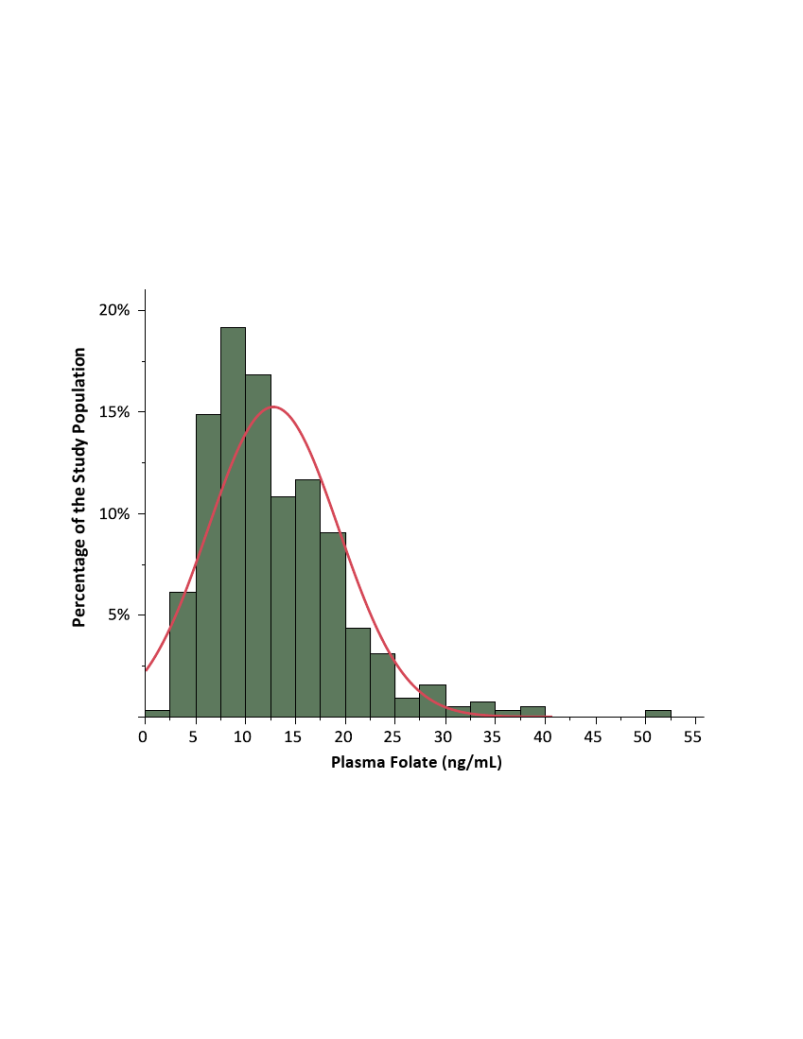

Supplement: Figure S6 — Distribution of plasma folate in the study population. (TIF) [file pone.0054544.s006.tif]

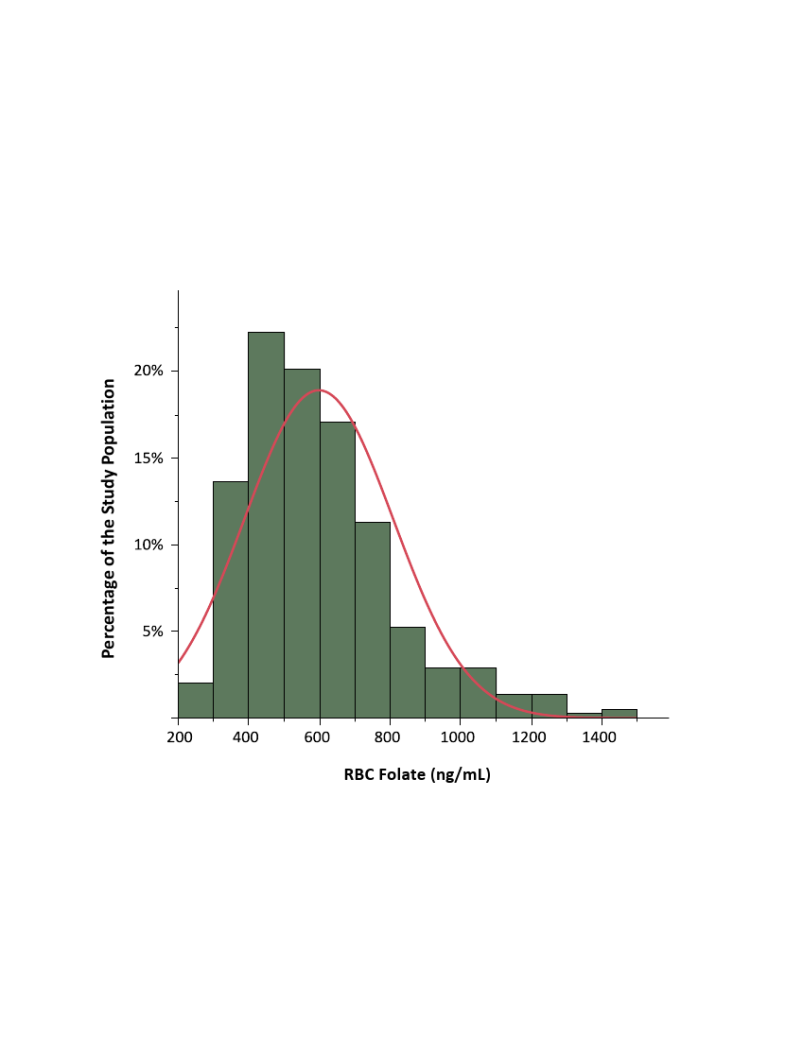

Supplement: Figure S7 — Distribution of RBC folate in the study population. (TIF) [file pone.0054544.s007.tif]

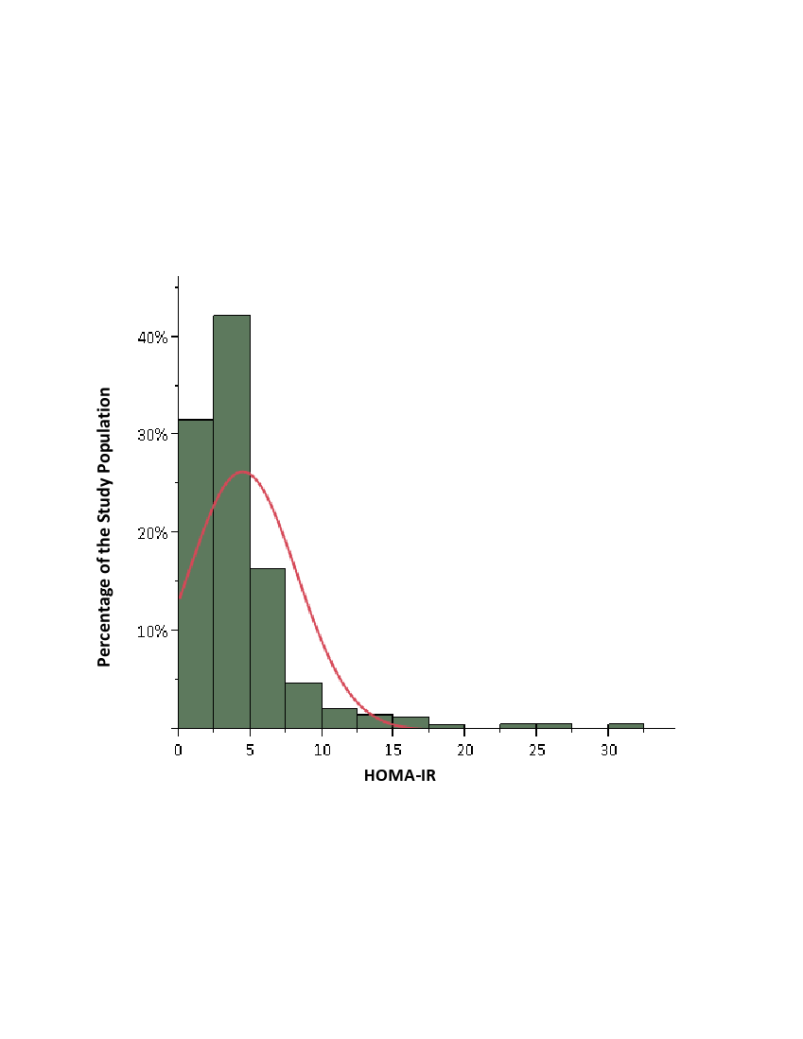

Supplement: Figure S8 — Distribution of HOMA-IR in the study population. (TIF) [file pone.0054544.s008.tif]
